# Quantifying the Effectiveness of Defensive Playing Styles in the Chinese Football Super League

**DOI:** 10.3389/fpsyg.2022.899199

**Published:** 2022-06-02

**Authors:** Lingfeng Ruan, Huanmin Ge, Yanfei Shen, Zhiqiang Pu, Shouxin Zong, Yixiong Cui

**Affiliations:** ^1^School of Sports Engineering, Beijing Sport University, Beijing, China; ^2^Institute of Automation, Chinese Academy of Sciences, Beijing, China; ^3^AI Sports Engineering Lab, School of Sports Engineering, Beijing Sport University, Beijing, China

**Keywords:** defense, multivariate regression, match analysis, xG, PCA

## Abstract

Establishing and illustrating a predictive and prescriptive model of playing styles that football teams adopt during matches is a key step toward describing and measuring the effectiveness of styles of play. The current study aimed to identify and measure the effectiveness of different defensive playing styles for professional football teams considering the opponent’s expected goal. Event data of all 1,120 matches played in the Chinese Football Super League (CSL) from the 2016 to 2020 seasons were collected, with fifteen defense-related performance variables being extracted. The PCA model (KMO = 0.76) output eight factors that represented 7 different styles of play (factor 6 and 8 represent one style of play) and explained 85.17% of the total variance. An expected goal (xG) model was built using data related to 27,852 shots. Finally, the xG of the opponent was calculated in the multivariate regression model, outputting five factors that (*p* < 0.05) explained 41.6% of the total variance in the xG of the opponent and receiving a dangerous situation (factor 7) was the most apparent style (31.3%). Finally, the predicted model with defensive styles correlated with actual xG of the opponent at *r* = 0.62 using the 2020 season as testing data which showed that the predicted xG was correlated moderately with the actual. The result indicated that if the team strengthened the defense closed to the own goal, high intensity confrontation, and defense of goalkeeper, meanwhile making less errors and receiving less dangerous situations, the xG of the opponent would be greatly reduced.

## Introduction

Football match performance incorporates the interactive effects of technical, physical, and tactical activities among players (Moura et al., 2013). Such interaction is conditioned by the strategical plans and match dynamics (Grehaigne et al., 1997) and can be explained by measuring the offensive and defensive behavior of teams and opponents (Carling et al., 2005). Consequently, various types of match approaches were adopted to integrate these dynamical interactions adopted by teams under distinct competition scenario (Fernandez-Navarro et al., 2016; Hewitt et al., 2016; Lago-Peñas et al., 2018). Hence, the tactical approach of a team in a particular match can be defined and depict how the football match unfolds (Lago-Peñas et al., 2018). Specifically, the term “style of play” refers to the dominant and recurring pattern demonstrated by a team in a specific competitive situation where the measurement of some performance indicators may reflect the team’s playing styles (Fernandez-Navarro et al., 2016; Hewitt et al., 2016; Zhou et al., 2021). Compared with “model of game” defining a complete technical and tactical process for the game (Sánchez et al., 2021) and “game philosophy” including the culture and ethos of a team (Fernandez-Navarro et al., 2016), the term “style of play” refers more to the behavioral pattern demonstrated by a team in a specific competitive context. Determining and measuring playing styles in elite soccer have a direct application into practice and competition (Kubayi and Toriola, 2020), such as modeling performance improvement in team’s strategies, player’s evolution, and scouting (Zhou et al., 2021).

Currently, the available research focusing on playing style in football has evolved in its approach measuring those key playing patterns which defined the styles of play. Hewitt et al. (2016) pointed out that a playing style pattern was represented by a team at five moments in a match: set offense, transition from offense to defense, set defense, transition from defense to offense, and set pieces. Fernandez-Navarro et al. (2018) classified playing styles of English Premier League into eight factors based on traditional techniques and tactics: direct play, counterattack, maintenance, build up, sustained threat, fast tempo, crossing, and high pressure. Yi et al. (2019b) defined three types of playing styles to characterize the matches of 2018 FIFA World Cup by a machine learning algorithm: direct play, possession play, and mixed play. Moreover, in the most recent studies, the statistical model is factor analysis (PCA: Principal Component Analysis) focusing on the identification of football playing styles (Gómez et al., 2018; Lago-Peñas et al., 2018; Zhou et al., 2021). The first study defined eight plying styles *via* selecting over sixty match performance variables in Greek professional football which jointly influencing how teams alternated their styles (Gómez et al., 2018). Meanwhile, both the second study (Lago-Peñas et al., 2018) and the recent study (Zhou et al., 2021) encountered different playing styles from the consideration of enough match performance indicators in Chinese football league. However, compared to the wide range of studies focusing on the attacking styles, few attempts have been done for the defense. Fernandez-Navarro et al. (2019b) tried to classify the defensive styles of play according to the zones of the pitch where the ball was regained, but not conducting deep research on the defensive styles.

In light of such phenomenon, quantifying the effectiveness of more styles of play has also been analyzed to explain a broader concept of tactical behaviors, to which these tactical variables and performance indicators contribute. Recent studies proposed a theoretical framework for measuring styles of play (Fernandez-Navarro et al., 2018), quantify the use of attacking and defensive strategies in football matches (Villa and Lozano, 2019), and predict a pattern of tactical–strategic behaviors with major probabilities of success in transitions (Casal et al., 2016). Behavior indicators (Kempe et al., 2014), multivariate statistical methods (Moura et al., 2014), and spatio-temporal analysis (Memmert et al., 2016) have also been used for potential tactical evaluation. In addition, new effectiveness metrics that take into account multiple variables have been developed recently. Expected goals (xG) is a metric used to assess the chance of a shot resulting in a goal which is useful for coaches and practitioners (Rathke, 2017). Fernandez-Navarro et al. (2019a) used xG as a metric to evaluate the effectiveness of playing styles in different contextual variables. Nevertheless, the effectiveness of playing styles in football is still inconclusive due to previous studies which were concentrated on an isolated attacking performance dimension (Liu et al., 2019). What’s more, previous studies have acknowledged that the defensive styles of play and performance indicators related to defense in different studies were not clear or comprehensive which might produce partially biased outcomes (Vogelbein et al., 2014; Bradley et al., 2016; Souza et al., 2019).

The lack of relevant studies evaluating the effectiveness of defensive styles could be attributed to the fact that most of the defensive events are recorded along with ball-related offensive behaviors, so that the tactical intention and the off-ball performance behind certain defensive technical actions may not be fully represented to produce reliable outcomes. Additionally, definitions and measurement of football defensive playing styles were varied among different studies. More styles of play should be considered when evaluating the defensive effectiveness. Therefore, the aim of the current study was: (i) to describe defensive playing styles in professional football *via* considering more comprehensive set of defensive actions and spatial information of teams, and (ii) to rate the effectiveness of different styles, accounting for opponent’s scoring opportunities (xG). It has been hypothesized that teams could be classified and evaluated according to their playing styles and the xG of the opponent could evaluate the effectiveness of defensive styles adopted by coaches.

## Materials and Methods

### Sample

The sample was composed of teams that played all 1,120 matches in the CSL from the 2016 to 2020 seasons (There are 160 matches of CSL in the 2020 season due to prevention and control of COVID-19). The Chinese Super League (CSL) is the top-professional soccer league in China, which starts in March (spring) and ends in November (winter) every season. Sixteen teams (*n* = 240 matches per season) play each other in a balanced schedule (each team plays against different opponents (home and away) twice). Match statistics were retrieved from the website of “Whoscored”,^1^ whose data were provided by OPTA Sports. The data provider has been previously verified to have high intra-class correlation coefficients (ranged from 0.88 to 1.00), low standardized typical errors (varied from 0.00 to 0.37) a, and very good strength of Cohen’s Kappa (>0.9) for inter-operator reliability of data collection (Liu et al., 2017).

### Performance Indicators and Procedure

**Figure 1:**
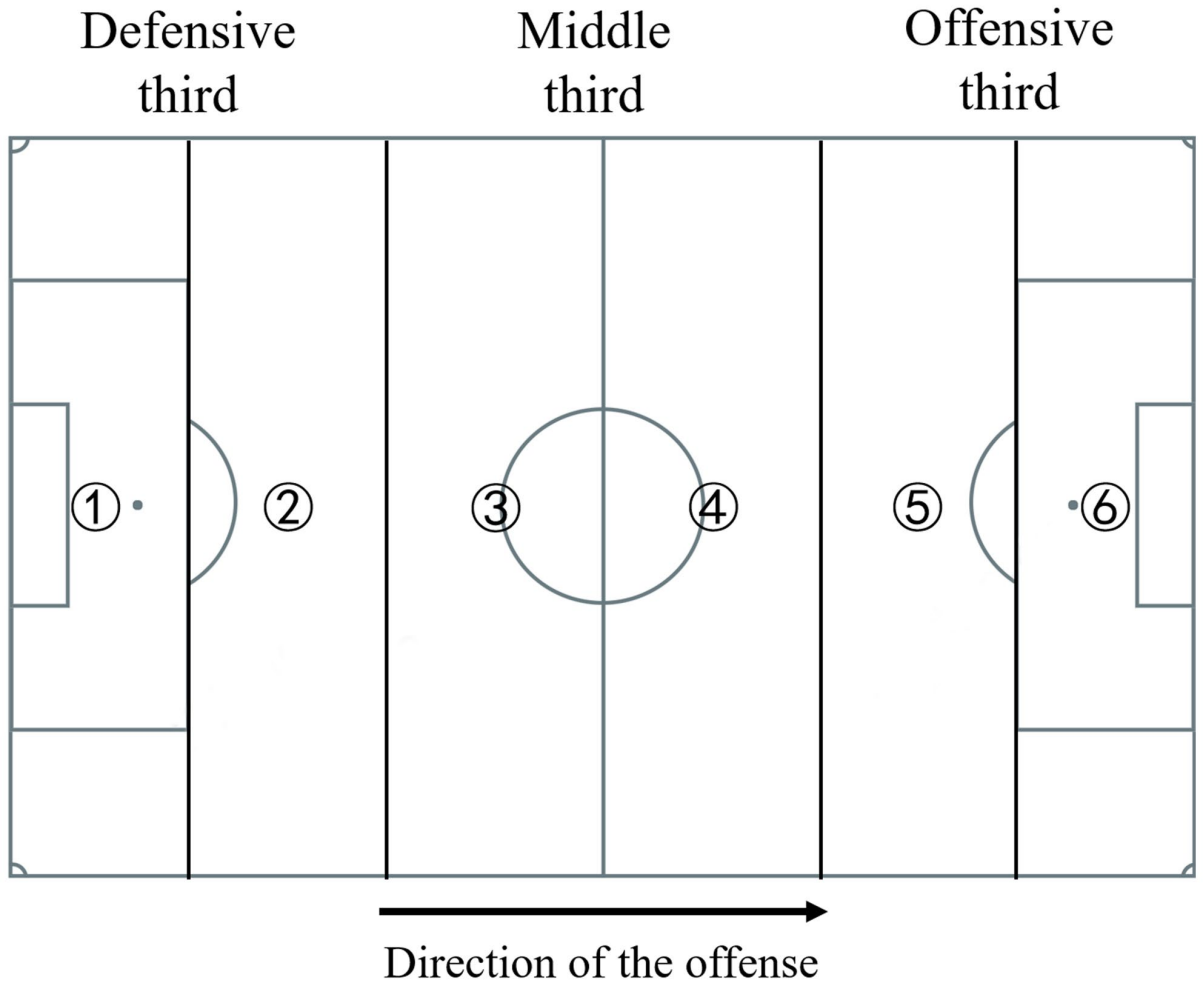
Pitch divisions in six zones parallel to the goal lines. ① represents zone 1; ② represents zone 2; ③ represents zone 3; ④ represents zone 4; ⑤ represents zone 5; ⑥ represents zone 6.

Based on the scientific literature that included defensive aspects in football match analysis (Yi et al., 2018, 2019a; Fernandez-Navarro et al., 2019b), a total of 15 defensive performance indicators related to technical and tactical performance were extracted from the raw data as dependent variables in the analysis due to their importance for measuring performance related to recovery the possession of the ball and match outcomes (Zhou et al., 2021). The description and definitions of defensive performance indicators are presented in Table 1. In addition, the pitch was divided into six spaces parallel to the goal lines for the extraction of the following performance indicators: pass in zone 3 and zone 4, and ball gain in the zone 1 (see Figure 1).

**Table 1:** Description and definition of the defensive performance indicators.

Variable	Definition
Related to own team	
1. Interception	A player reads an opponent’s pass and intercepts the ball by moving into the line of the intended pass. The interception could be finished with or without the ball recovery
2. Clearance	A player kicks the ball away from his own goal with no intended recipient, and the clearance included that the ball kicked from the offensive filed or kicked to the sideline
3. Ball gain zone 1	The analyzed team gained the ball in zone 1 (see Figure 1)
4. Error total	The number that a defensive player makes total errors, which leads to goals or shots conceded
5. Error in own half (%)	The number that a defensive player makes errors in own half divided by total errors
6. Keeper claim	The number of times that the goalkeeper get possession of the ball positively
7. Keeper smother	The number of times that the goalkeeper who comes out and claims balls at the feet of a forward gets smothers, similar to tackles
8. Foul total	The number of times that a player commits a foul for defense
9. Creating danger	After gaining the ball, the analyzed team made a shot or entry into the opposing penalty area
Related to opponent team
10. Deep completion	The number of pass (excluding crosses) that was received in a 20-meter radius from the opponent goal line
11. Cross unsuccess	The number of teams crossed unsuccessfully
12. Dribble success	The number of teams dribbled successfully
13. Shot accuracy (%)	The number that a player shot accuracy
14. Pass in the zone 3	Any kind of pass made by the team in zone 3 (see Figure 1). And the of the pass is in the designated zone 3
15. Pass in the zone 4	Any kind of pass made by the team in zone 4 (see Figure 1). And the of the pass is in the designated zone 4

**Figure 2:**
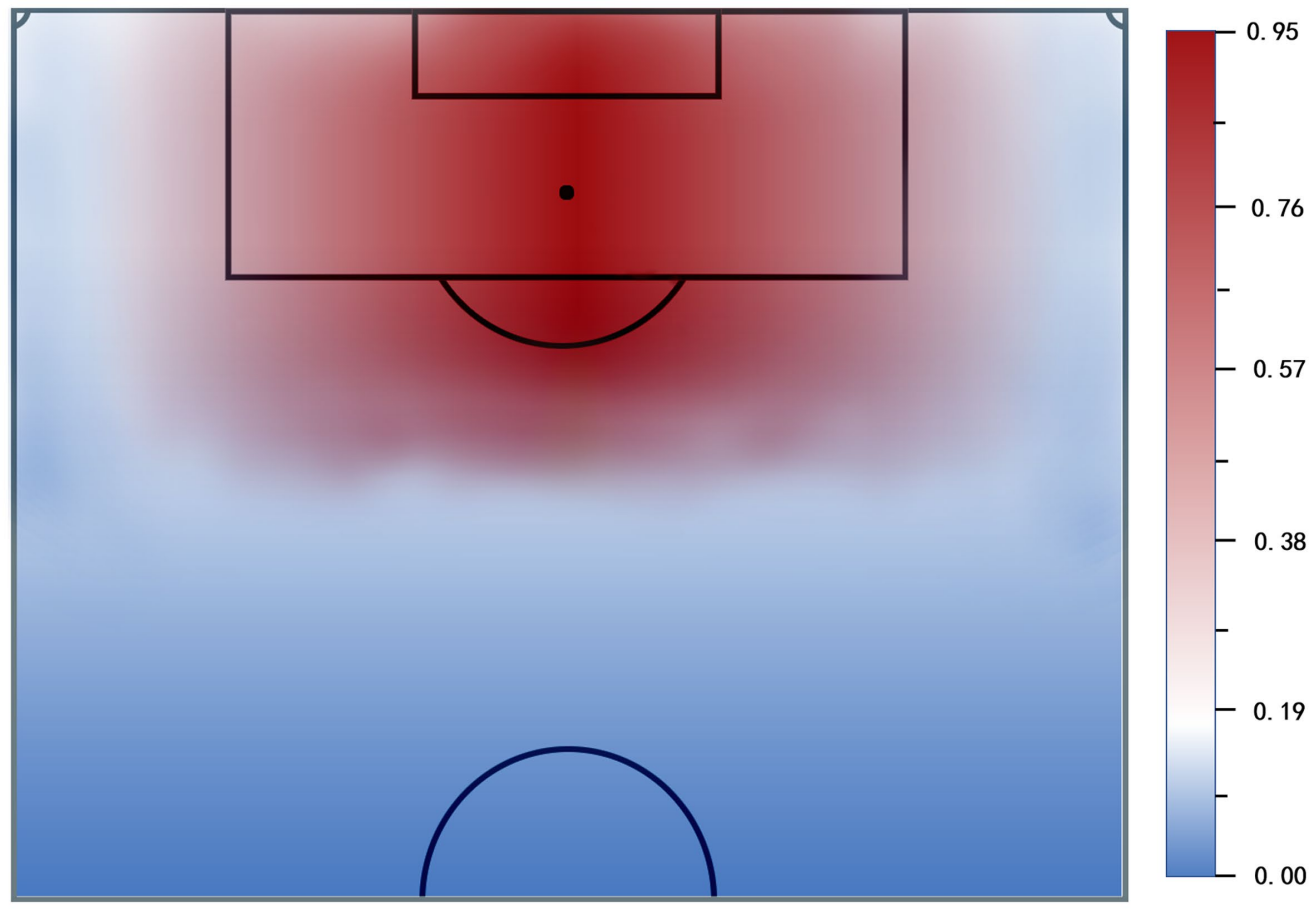
Expected conversion probabilities of shots on the pitch. The darker the red, the higher the expected goal.

Expected goals (xG) measures the conversion probability of a shot based on pitch location and types of start (e.g., cross and counterattack) and finish (e.g., shot and headed shot). The xG assigns a quality value ranging from 0 to 1 for each shot toward the goal (scored or not scored) with a higher value indicating a greater likelihood of a scoring opportunity. For instance, a headed shot from the central position on the edge of the six-yard box has an xG value of 0.669. In other words, head shots taken from this position would have 66.9% probability of scoring a goal. The study regarded the xG of opponent teams as the reference to evaluate the effectiveness of each defensive playing style. The expected goal (xG) of the opponent team with a lower value in a match meant that the defensive team had a better performance in recovering the ball or preventing the opponent from scoring a goal. On the contrary, the defensive team with a higher xG of opponent teams had less effect on preventing offense through technical and tactical actions of defense. Therefore, the study extracted a total of 16 features related to offensive and defensive performance from the raw data based on the previous studies (Bransen et al., 2019; Yi et al., 2020; Haaren, 2021) to construct the xG model: X position of shot, Y position of shot, body part, counterattack, shooting distance to goal line, shooting distance to center, whether using the weak foot, shooting by head distance to goal line, last action prior to the shooting (cross, corner, key pass, dribble, and duel), angel of shooting, free kicks, and penalties. Finally, the distribution of xG values were plotted in the following figure (Figure 2).

### Statistical Analysis

Firstly, exploratory factor analysis using principal component analysis (PCA) was conducted on the defensive performance indicators with orthogonal rotation (varimax) running with all 15 performance indicators in order to pool the variables into factors (dimensions of defensive playing styles). This technique allowed the data set to be reduced to factors by grouping the measured variables (Field, 2013). To facilitate the comparison of teams’ defensive performance in each factor, the scores were later normalized to unify different scales of all factors. The normalized factor score for each team was obtained *via* dividing the original factor score by the median. For each factor, the performance indicators with the highest factor loading (i.e., the correlation between the performance indicator and the factor) are identified. This technique pools performance indicators into fewer factors that represent different styles of play.

The Kaiser–Meyer–Olkin measure (Kaiser, 1974) and communalities values after extraction (Maccallum et al., 1999) were employed to verify the sampling adequacy for the analysis. The adequacy of inter-item correlations is based on Bartlett’s sphericity test. Performance indicators with factor loadings greater than |0.6| showed a strong positive or negative correlation and indicated a substantial value for factor interpretation (Daniel, 1993).

Secondly, a xG model (a machine learning model—logistic regression analysis) was calculated using 27,852 shots from five seasons of CSL data (2016–2020). In order to improve the interpretability of the result and the accuracy of the model, there were more performance indicators related to shots were chosen as independent variables in the model. The metric of xG model was called AUC. The AUC of a model is the probability that the model ranks a random positive example (i.e., a successful shot) more highly than a random negative example (i.e., unsuccessful shot). For instance, 73.32% (AUC) meant that when randomly selecting a successful shot and an unsuccessful shot, there was a 73.32% chance that the model gave the successful shot a higher probability than the unsuccessful shot. Thus, this provided the basis for the expected goals model to calculate the likelihood of a shot resulting in a goal. A detailed explanation of the xG model and multiple performance indicators that covered this metric could be found in the study by Bransen et al. (2019) and Haaren (2021).

Lastly, the study performed a multiple regression analysis in a stepwise interactive mode in order to assess the influence that each defensive playing style had on the effectiveness of defense. The team’s score of each factor in a match was used as an independent variable. And xG of the opponent team in the same match was a dependent variable. The training model was calculated from the 2016 to 2019 seasons. And the 2020 season was used for a testing model to measure accuracy and validity of the multiple regression model. When testing the multiple regression model, Pearson’s correlation analysis of the predicted xG and the real xG was conducted. And the correlation coefficient (*r*) was calculated with absolute values of the thresholds being 0- to 0.3-weak correlation, 0.3–0.5 low correlation, 0.5–0.8 moderate correlation, and 0.8–1.0 high correlation (Field, 2013). To check the normality of the data, the Kolmogorov–Smirnov test was used. The homogeneity of variance was tested by Levene’s test. This analysis was inspired by similar investigations in professional football leagues (Aquino et al., 2017; Souza et al., 2019). In the regression analysis, all match statistics were introduced based on their correlation with the residual and their intercorrelation with variables that already included in the equation. Redundant variables were excluded to avoid multicollinearity by using the variance inflation factor (VIF). The $R^{2}$ values were adjusted for the number of cases and parameters in the analysis. Using the standardized regression coefficients, the relative contribution of each different variable in relation to the explained variances was calculated as follows:


(1) $$R_{adjusted}^{2}=\frac{B_{standardized}}{B}$$

where $R_{adjusted}^{2}$ = partial contribution $R^{2}$ adjusted of a style, $B_{standardized}$ = standardized regression coefficient for parameter, B = sum of all standardized regression coefficients in equation.

All the analyses were performed using Python 3.6 and statistical significance level was set at *p* < 0.05.

## Results

The Kaiser–Meyer–Olkin (KMO) measure verified the sampling adequacy for the analysis with a score of 0.76, and the communalities after extraction were greater than 0.6 in all performance indicators, deeming sample size to be adequate for factor analysis. Bartlett’s test of sphericity ( $\chi^{2}$ =3,258, df = 105, *p* < 0.05) indicated that correlations between items were sufficiently large for PCA. Eight components had eigenvalues (rotation sums of squared loadings) over one and explained 85.17% of the total variance. The percentage of variance explained by each factor decreased from factor 1 to 8. The rotated component matrix for the factor loadings determined the performance indicators associated with each factor (Table 2).

**Table 2:** Rotated component matrix for the performance indicators showing a strong positive or negative correlation.

	Component							
	1	2	3	4	5	6	7	8
Interception			0.625					
Clearance	0.888							
Ball gain zone 1	0.875							
Error total				0.970				
Error in own half				0.974				
Keeper claim								0.986
Keeper smother						0.948		
Deep completion	0.746							
Foul total			0.622					
Cross unsuccess	0.833							
Dribble success		−0.687						
Shot accuracy							0.943	
Pass in the zone 3		−0.810						
Pass in the zone 4		−0.876						
Creating danger					0.799			

Descriptions of factors were interpreted based on the group of associated performance indicators. A new factor score obtained after the team’s factor score was normalized according to the median determining how much a team relies on one specific defensive style or a combination of these styles of play. Factor 1 (Defense closed to the own goal) included clearance, ball gain zone 1, deep completion, and cross unsuccess. A team with a high score on this factor tended to defend in the defensive third. Factor 2 (mid-positioning defense with pressure) included dribble success, pass in the zone 3 and pass in the zone 4. The team that scored positively had a lower percentage of possession in the central third. Factor 3 (High intensity confrontation) included interception and foul total. A team with a high score regained more balls by fouling and dueling. Factor 4 (Error) defined teams that tended to makes an error leading to a goal or shot conceded if they scored highly. Factor 5 (Defense in advanced zones) defined teams creating a dangerous situation after giving more pressure in the offense third. Factor 6 and factor 8 (Defense of goalkeeper) identified the importance of keeper smother and keeper claim. Finally, factor 7 (Receiving a dangerous situation) defined teams with a high score which was not so good at defending that the opponent teams made dangerous situations frequently.

**Figure 3:**
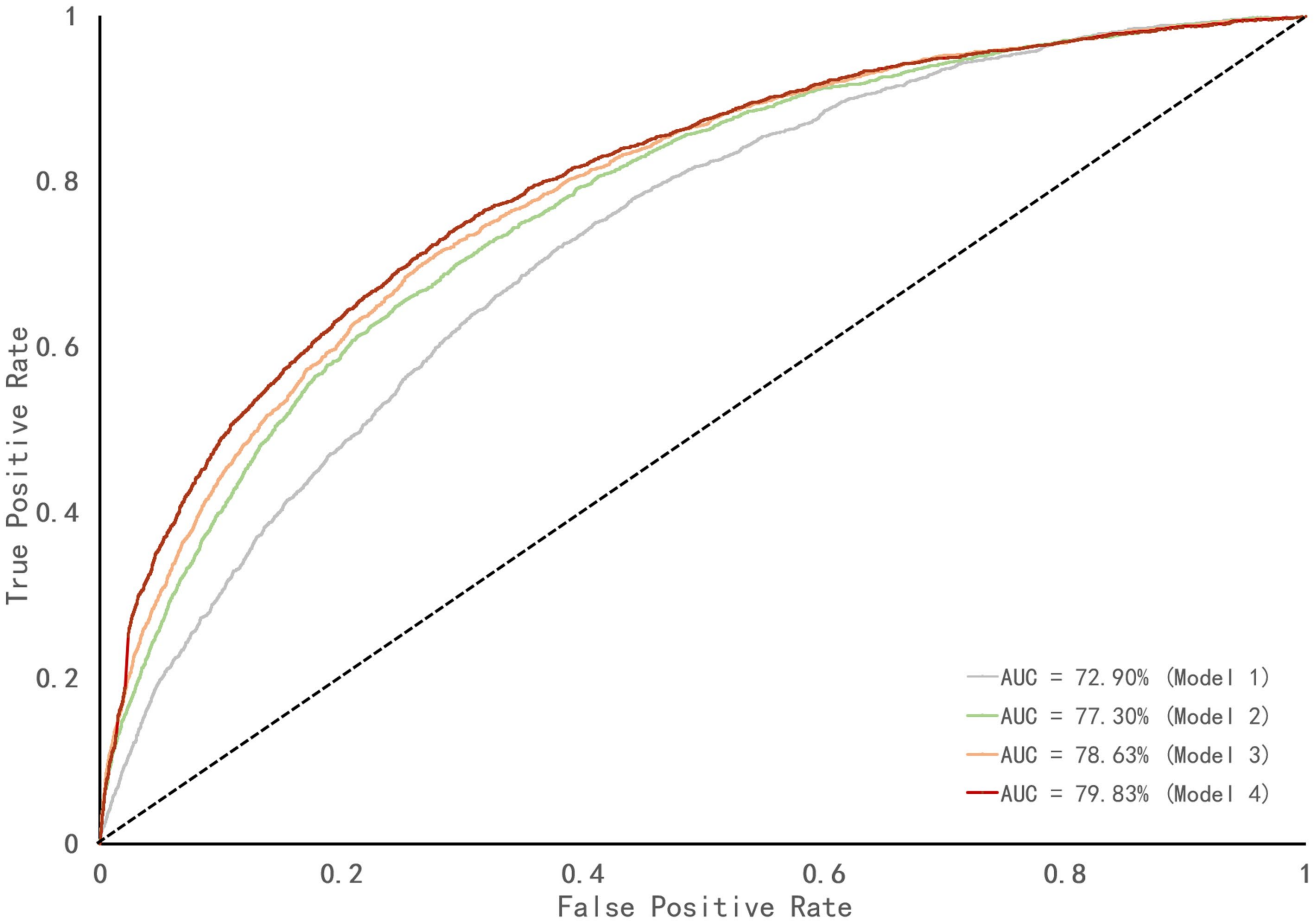
The ROC curve of different logistic regression models in xG. The diagonal dotted line represents a line of zero discrimination, also known as pure chance line.

The process of improving the ROC curve of the models in xG was showed in Figure 3, with the value of AUC increasing from model 1 (AUC = 72.90%) containing X position of shot, Y position of shot, body part, and counterattack to model 4 (AUC = 79.83%) including free kicks, and penalties constantly, showing that when randomly selecting a successful shot and an unsuccessful shot, there was a 79.83% chance that the model gave the successful shot a higher probability than the unsuccessful shot.

**Figure 4:**
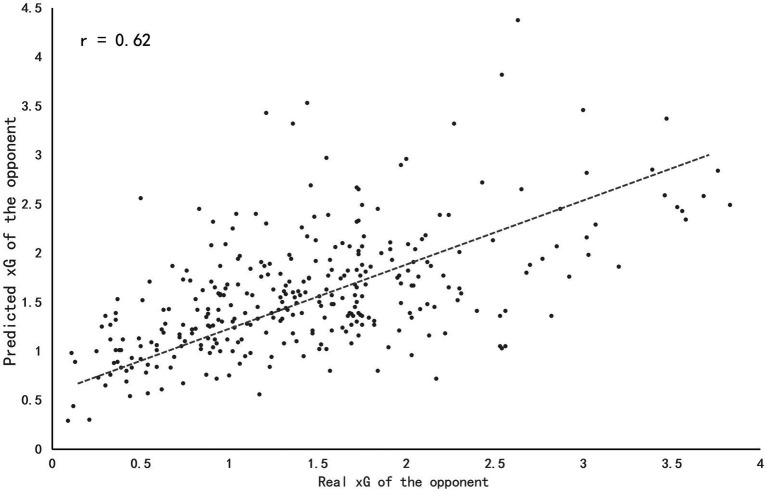
Correlation between the actual and predicted xG of the opponent of 2020 season in CSL. Each dot represents one team in a match, for a total of 320 real-predicted comparisons. The dotted line represents the trend of correlation between the actual and predicted xG of the opponent.

Table 3 summarized the results of the multiple linear regression. Regarding degree of importance, the factor 7 (receive a dangerous situation) explained 31.3% of the variance in xG of the opponent in the model. Adding the factor 1 (defense closed to the own goal), this percentage reached 38.9%. Factor 4 (error) and factor 8 (keeper claim) explained 1.4 and 1% of the variance in the model. Finally, the styles of play adding the factor 3 (high intensity confrontation) explained 41.6% of the total variance in the model. On the contrary, factor 2 (mid-positioning defense with pressure), factor 5 (defense in advanced zones), and factor 6 (keeper smother) were excluded from the multiple regression model of prediction (all *p* > 0.05). Overall, the multiple regression analysis explained 41.6% of the total variance in the xG of the opponent. In this case, factor 7 (receive a dangerous situation) was the defensive style of play with the highest association to the xG of the opponent. Figure 4 included a comparison between the predicted and actual xG of the opponent with the multiple regression model of prediction by using the data of 2020 season. The predicted xG of the multiple regression model correlated with actual xG of the opponent at *r* = 0.62 (*p* < 0.05) showing a moderate correlation.

**Table 3:** Relative contribution of defensive styles of play to the variance of excepted goals (xG) of the opponent frequency.

Predictors	Non-standardized β coefficients	Standardized β coefficients	Adjusted $R^{2}$	*t*	*p*	Tolerance	VIF
Constant	0.219			2.561	0.01^*^		
Receiving a dangerous situation	2.405	0.917	0.312	31.872	<0.01^**^	0.68	1.46
Defense closed to the own goal	−1.053	−0.441	0.389	−15.375	<0.01^**^	0.61	1.64
Error	0.155	0.109	0.403	6.216	<0.01^**^	0.60	1.66
Keeper claim	−0.040	−0.101	0.413	−5.688	<0.01^**^	0.58	1.71
High intensity confrontation	−0.175	−0.051	0.416	−2.923	<0.01^**^	0.58	1.72
Mid-positioning defense with pressure	−0.021			−1.042	0.30	0.79	1.27
Defense in advanced zones	−0.042			−1.946	0.05	0.65	1.54
Keeper smother	0.009			0.497	0.62	0.99	1.02

VIF, Variance inflation factor. Adjusted *R*^2^ is cumulative, with each incremental step adding to the variance explained.

* *p* < 0.05;

** *p* < 0.01.

### Predictive Model of xG of the Opponent Based on Defensive Styles of Play

Expected goals of the opponent = 0.219 + (receive a dangerous situation × 2.405) + (defense closed to the own goal × − 1.053) + (error × 0.155) + (keeper claim × − 0.040) + (high intensity confrontation× − 0.175) (2).

## Discussion

The main aim of the current study was to identify and measure defensive playing styles used by the CSL teams during a five seasons period (from the 2016 to 2020 seasons) *via* considering a more comprehensive set of defensive techniques and spatial information; and to investigate the effectiveness of defensive styles of play during the seasons under analysis. As it was hypothesized, this study identified specific defensive playing styles based on fifteen technical–tactical indicators that varied along the matches as an adaptation of teams in various situational variables to perform at the highest level in the competition. To the best of our knowledge, although some investigations have described partially different styles of play in elite soccer (Fernandez-Navarro et al., 2016; Gómez et al., 2018; Lago-Peñas et al., 2018), no previous study has examined the effectiveness of the defensive playing styles used by teams and their association with the success of defense.

The current study identified 8 factors from the PCA: factor 1 (defense closed to the own goal), factor 2 (mid-positioning defense with pressure), factor 3 (high intensity confrontation), factor 4 (error), factor 5 (defense in advanced zones), factor 6 (keeper smother), factor 7 (receive a dangerous situation) and factor 8 (keeper claim). Meanwhile, the PCA results showed scores of 8 factors for teams indicating their reliance on specific defensive styles of play. Compared to the study of Castellano and Pic (2019) that classified playing styles of the Spanish first division (LaLiga) into two offensive phases (direct attack versus elaborate attack) and two defensive phases (deep defending versus high-pressure defense), the current study is able to achieve a complex classification of defensive styles *via* including the spatio-temporal and zonal information of more defense performance indicators. Such approach could therefore facilitate the assessment of defensive effectiveness. Moreover, these findings might not only allow analysts to identify their own team’s defensive playing styles in order to recognize their own strengths and weaknesses, but also inform coaches when designing specific trainings where teams are required to play against simulated opponents of different defensive styles or to strive to defend with the most efficient style (Díaz-Díaz et al., 2019).

The accuracy of the logistic regression model for xG was improved (AUC = 79.83%) after the training process, and the variables have considerably high interpretability for explaining offensive behaviors. Meanwhile, the predicted xG by the multiple regression model were consistent with actual xG (*r* = 0.62). Defense closed to the own goal (factor 1), high intensity confrontation (factor 2), error (factor 4), receive a dangerous situation (factor 7) and factor 8 (keeper claim) were found to be the most important variables of the model, implying that teams with the above styles achieved greater efficiency in preventing opponents from scoring. Interestingly, factor 7 was the variable that explained more proportion of the variance, and it is defined as whether the goal was threatened after teams losing possession of the ball. Higher scores in this variable could mean that teams were slow in transition so that it created a disadvantageous situation for themselves. Such claim is supported by the previous finding that shot accuracy (one of the variables in factor 7) could differentiate the best-ranked and least-ranked teams in LaLiga (Lago-Ballesteros and Lago-Peñas, 2010). Therefore, it is crucial for the defending team to be fast in transition once losing the possession and try to undermine the opponent’s offensive *via* forcing them to make non-threatening passes (Ali, 2011; Kempe et al., 2014). In addition, error (factor 4) including error and error in own half was also positive with xG of the opponent, which suggested that teams had higher scores in this factor tended to commit mistakes so frequently that the possession was easily lost. Previous research showed that ball recoveries closer to the attacking goal produced seven times more goals and 19 times more entries into the penalty area (Yi et al., 2019a), compared to ball recoveries in the defensive zones. Therefore, it was actually rather essential for defensive teams to make less errors especially in their own half.

Besides, factor 1 (defense closed to the own goal), factor 3 (high intensity confrontation) and factor 8 (keeper claim) were negative with xG of the opponent in the multiple regression model. Defense closed to the own goal (factor 1) explained the highest percentage of variance among them, while high intensity confrontation (factor 3) higher than factor 8. These defensive styles of play occupying a large proportion may be due to two reasons. On the one hand, it is widely believed that the growing presence of non-Chinese players in the CSL could account for these alterations in technical, tactical, and confrontational performance. In fact, it was plausible for non-Chinese players to encourage Chinese players to improve their physical and technical performance. Bush et al. (2015) found that the English Premier League has underwent substantial changes over the last decade with the distances of high intensity and sprinting increasing by 30–50% and the number of passes rising by 40%. On the other hand, it may be due to their traditional playing tactics for most CSL teams: dropping the line to avoid losing the ball and waiting for the chance to counterattack which might reduce xG of the opponent. It suggested that the tactical principle of counterattacking caused imbalances in the opposition’s defense and offense, therefore increasing the success of the attacking sequence and the chance to score a goal (Tenga et al., 2010). As for keeper claim (factor 8), it suggested that goalkeepers scoring highly tended to get possession of the ball positively which led to reduce xG of the opponent. In the modern football matches, goalkeepers were required not only to defend the goal, but also to actively cooperate with their teammates both during defending and attacking as an organizing role (Hughes and Bartlett, 2002; Dellal et al., 2011). Szwarc et al. (2019) who showed that the goalkeepers in the bottom 5 teams have a higher distance of sprint compared to the top five teams in the English Premier League.

In contrast, factor 2 (mid-positioning defense with pressure), factor 5 (defense in advanced zones), and factor 6 (keeper smother) were excluded from the regression analysis (*p* > 0.05). Mid-positioning defense with intense pressure (factor 2) suggested that the team which scoring lower paid more defensive attention to and put more pressure on the middle third so that the team gained much possession in the middle third and forward players had time and space to come into the box and seize the opportunity to shot. And defense in advanced zones (factor 5) identified teams that used high- or low-pressure defensive styles of play in the advance zones. Defense in advanced zones could influence scoring opportunities as the ball could be recovered closer to the opponent’s goal and increased the likelihood of facing an imbalanced defense (Wright et al., 2011). To sum up, concentrating more on the middle third suggested a possession style, while putting more pressure on the attacking third suggesting a high-pressure style. Nevertheless, there were two reasons for the two styles of defense being excluded. On the one hand, in the CSL, there were few teams having their playing philosophy for high-pressure and the highly skilled middle players in the team for passing abilities to have an impact on xG of the opponent where progress and breakthroughs should be made in the future for CSL teams. These findings were in accordance with available research suggesting top teams prefer to “control” the game by dictating it instead of giving the initiative to the opponent to protect own goal (Collet, 2013). On the other hand, these outcomes suggested that a more vertical style of play, where shot finalization on the goal became the main offensive objective, may be a more successful strategy to succeed in football instead of the prevalent idea of maintaining ball possession and passing over the opponent (Souza et al., 2019). As for keeper smother (factor 6), this was mainly because the defense was closer to the midfield and the forward which resulted in smothering for goalkeepers when the opponent got a chance of a single-pole ball. And realizing successful smother was too difficult for goalkeeper to influence xG of the opponent.

With regard to the limitations of the present study, some aspects should be highlighted to improve the applicability of its results. Firstly, the multiple regression analysis explained relatively low percentage (41.6%) of the total variance in the xG of the opponent. Such result could be due to two reasons. On the one hand, using xG of the opponent alone might not be able to comprehensively measure the defense of teams, as the former was highly relevant to the opposing offense. On the other hand, the physical, technical–tactical, and positional related variables concerning off-ball actions were not included in the current research, and should be considered in the future (Liu et al., 2015; Zhou et al., 2021). Secondly, the analysis of interactive effect of contextual-related variables (e.g., in-home vs. visitor; match status—winning, losing, or drawing; and the moment of match—begin of championship, middle, and end) need to be addressed to determine their impact on the selection of defensive indicators and the effectiveness of defensive playing styles. In fact, previous studies have emphasized the importance of situational variables in assessing offensive performance (Fernandez-Navarro et al., 2018; Zhou et al., 2018), but not during the defensive phase. Lastly, different countries and competitions should be analyzed in order to verify the generalizability of the findings base on the CSL.

## Conclusion

In summary, this study allowed to identify and measure eight factors that represent seven different styles of play from fifteen defensive performance indicators based on a more comprehensive set of defensive techniques and spatial data by the PCA model. The team’s ranking showed different performance trends according to each team. After selecting these defensive styles of play and xG of the opponent to run the multiple regression model, five defensive styles were identified to have an influence on the effectiveness of defense in the CSL. If the team strengthened the defense closed to the own goal, high intensity confrontation, and defense of goalkeeper, meanwhile making less errors and receiving less dangerous situations, the discipline for opposing team to shoot and score would be greatly reduced. According to their team’s effectiveness and efficiency of defensive styles, coaches can deploy specific tactics and teams can choose appropriate players in the player markets. Further research should attempt to establish the influence of situational variables and off-ball actions on defensive style when measuring performance and outcomes.

## Data Availability Statement

Publicly available datasets were analyzed in this study. This data can be found at: www.whoscored.com.

## Author Contributions

LR, SZ, and YC: conceptualization and data curation. LR and YC: formal analysis. LR, HG, SZ, and YC: investigation. LR, HG, YS, ZP, SZ, and YC: methodology, software, writing— original draft preparation, designing the experiments and performing the statistical analysis, writing—review and editing, and writing and revising the manuscript. LR, ZP, and YC: visualization. YS, ZP, and YC: funding acquisition. HG, YS, ZP, SZ, and YC: supervising the design and reviewing the manuscript. All authors have made a substantial and direct contribution to manuscript and approved the final version of the manuscript.

## Funding

This work was supported in part by the National Key Research and Development Program of China under grant 2020AAA0103404 and by National Natural Science Foundation of China under grants 72071018 and 72101032. YC was supported by the Fundamental Research Funds for the Central Universities of China (2021TD008).

## Conflict of Interest

The authors declare that the research was conducted in the absence of any commercial or financial relationships that could be construed as a potential conflict of interest.

## Publisher’s Note

All claims expressed in this article are solely those of the authors and do not necessarily represent those of their affiliated organizations, or those of the publisher, the editors and the reviewers. Any product that may be evaluated in this article, or claim that may be made by its manufacturer, is not guaranteed or endorsed by the publisher.

## Footnotes

^1^ www.whoscored.com
